# Trajectories of Television Watching from Childhood to Early Adulthood and Their Association with Body Composition and Mental Health Outcomes in Young Adults

**DOI:** 10.1371/journal.pone.0152879

**Published:** 2016-04-20

**Authors:** Joanne McVeigh, Anne Smith, Erin Howie, Leon Straker

**Affiliations:** Department of Physiotherapy and Exercise Science, Curtin University, Perth, Western Australia, Australia; Vanderbilt University, UNITED STATES

## Abstract

**Introduction:**

Prior studies examining longitudinal patterns of television (TV) watching have tended to use analytical approaches which do not allow for heterogeneity in the variation of TV watching over time. In the current study, we used latent class analysis (LCA) to examine the relationships between television watching (from childhood to early adulthood) and body fat percentage (%) and mental health.

**Methods:**

Data were collected from 2411 participants (50% female) from the Raine Study, a prospective birth cohort study in Australia. Participants were followed up over 15 years and answered questions about hours of TV watching per week at six time-points (5, 8, 10, 14, 17 and 20yrs). Trajectories of television watching were estimated using LCA and appropriate regression models used to test the association of television watching class with percentage body fat (measured by DXA) and mental health (DASS-21) at age 20. Physical activity was used as a covariate.

**Results:**

Three distinct trajectories of TV watching were identified. Class 1 (47.4%) had consistently high (>14 hrs/wk) levels of TV watching, Class 2 (37.9%) was characterised by an increase in TV watching over adolescence and Class 3 (14.7%) had consistently lower (<14 hrs/wk) TV watching over 15 years. Sex was used as an active covariate in the latent class model and was significantly associated with class membership (p<0.001), with females comprising 45%, 47% and 59% of Class 1, 2 and 3 respectively. In females, membership in Class 2 or 3 was associated with lower body fat % at age 20, compared to Class 1 (p<0.001). For males, membership in Class 2 was associated with lower body fat % compared with males in Class 1 (p = 0.026). Membership of TV watching class and mental health were not related (p>0.05).

**Conclusions:**

TV watching from childhood to young adulthood appears to be a relatively stable behavior for around two thirds of participants, but not everyone tracks consistently. This study identified a subset of participants with low levels of TV watching in childhood and also that this group, despite an increase in TV watching over adolescence, maintained a lower level of body fat in young adulthood.

## Introduction

Television (TV) watching is a prevalent behaviour across the life span and often is associated with the use of other screen based media and broader sedentary behaviour exposure [1]. In spite of the accumulated data from cross sectional studies showing the negative association of TV watching and various health outcomes, specifically at life-course epochs of early childhood [2, 3]; adolescence [4, 5]; young adulthood [6] and during later adult years [7], surprisingly little is known about the longitudinal pattern of change of TV watching over critical developmental periods [8] and how this may relate to early adulthood health outcomes.

Evidence from observational and experimental studies support a direct link between TV watching and increased body fat in children [9], adolescents [10] and in adults [11] and more recently an association has been described with poor mental health outcomes [12, 13]. However, only three studies have investigated the long-term effects of childhood TV watching on adult body mass index (BMI), reporting childhood TV watching (between 5 and 15 years) to significantly predict higher BMI in adulthood [14–16]. Although longitudinal assessments relating TV watching with the incidence of mental disorders are scarce, there is some evidence from longitudinal studies in adults, that the risk of depression may be slightly increased with increasing TV watching time [17–19], and that time spent in MVPA may attenuate the relationship [13]. To the authors’ knowledge no studies have investigated the longitudinal associations of TV watching in childhood with early adulthood mental health outcomes.

Although TV watching appears to be a fairly stable behaviour over a number of years of sample follow-up [14, 20, 21], few studies have data spanning more than five years. Further, studies which have discussed the tracking of TV watching over more than one time point have been limited in their analytical approach. Most have tended to only report a single tracking coefficient [14, 22] or have used generalized estimating equations [21, 23] to describe a single sample trajectory. These analyses assume all individuals follow the same pattern of change over time and do not allow for the modelling of population heterogeneity [24]. As such these analyses may oversimplify the potentially complex longitudinal patterns of TV watching, thereby missing identification of potential windows of opportunity for interventions to reduce TV watching exposure.

Latent class analysis (LCA) is a method that can be used for the exploration and identification of patterns of TV watching behaviours over time. Class membership is based on groupings of similar patterns of responses over time, and covariates can be used to predict class membership probabilities [24]. To the authors’ knowledge, only one other study has used a trajectory based approach to describe TV watching time over a longitudinal follow up from childhood to early adulthood [25]. However, the authors did not relate the TV trajectories to any health outcomes.

Given the health effects of excessive TV watching identified for both children and adults, a detailed understanding of the behaviour is needed. The purpose of this study was to report on the application of LCA to identify TV watching trajectories from childhood (5 years) to early adulthood (20 years). Furthermore, to add internal validity to the trajectories identified we explored the associations of these trajectories with physical and mental health outcomes in early adulthood. We also investigated whether physical activity had a moderating effect on the relationship between TV watching, body fat and mental health.

## Methods

### Participants

Participants for this study were part of The Western Australian Pregnancy Cohort (Raine) Study [26]. Briefly, 2900 pregnant women attending the public antenatal clinic at King Edward Memorial Hospital, or nearby private practices, were recruited into the Raine Study between May 1989 and November 1991. A total of 2868 children have undergone serial assessment at birth and at ages 1, 2, 3, 5, 8, 10, 14, 17 and 20 years. Informed, written consent to participate in the study was obtained from the mother of each child at enrolment and at each subsequent follow-up until the children achieved majority and provided their own consent. The study protocols have all been approved by the Human Research Ethics Committee of Curtin University (HR 23/2013) and Human Research Ethics Office the University of Western Australia (RA/4/1/5202).

At the 20-year follow up, 1565 participants took part. Complete data for TV watching, body fat, mental health and physical activity were available for 58% (n = 910) of the participants at age 20. Those included in the present study were not different for sex, BMI at age 5, family income at age 5 or income at age 20, but did have a slightly lower BMI at age 20 (p = 0.03), compared to the participants included in the 20-year follow up but for whom there were incomplete data for inclusion in this study (S1 Table).

### Television watching

At ages 5, 8 and 10, parents reported on the length of time their child spent watching TV per day using a six category question with response options from ‘none’ to ‘more than three hours’ per day. At ages 14, 17, and 20, study participants themselves responded to five category questions about the length of time they usually spent watching TV per day with response options from ‘none at all’ to ‘4 hours or more’ per day. Data were combined to create overall TV watching categories across all 6 time points of: i) no hrs/week, ii) less than 7 hrs/wk, iii) between 7 and 14 hrs/wk, iv) between 14 and 21 hrs/wk and v) more than 21 hrs/wk.

### Body Composition measures

At age 20, percentage body fat (and lean body mass) was obtained from a whole body Dual energy X Ray Absorptiometry (DXA) scan (Norland XR-36 densitometer, Norland Medical Systems, Inc., Fort Atkinson, WI, USA) performed according to manufacturer-recommended procedures using built-in software (version 4.3.0). All analyses were checked for consistency and daily calibration was performed on the DXA machine prior to each scanning session. The densitometer had a variation in precision of <2.0% for the measured site at standard speed. Weight was measured to the nearest 100g using Personal Precision scales UC-321 (A and D Company) and height was measured to the nearest 0.1 cm with a wall mounted stadiometer (Seca 202).

### Mental health

Mental health was assessed using the 21-item self-reported Depression Anxiety Stress Scales (DASS-21), which consists of three 7-item self-report scales that assess symptoms of depression, anxiety and stress [27]. The DASS-21 (and its longer form) have been validated in clinical and non-clinical populations [28, 29]. Participants were asked to rate the extent to which they had experienced each state over the past week on a four-point severity/frequency scale with responses ranging from 0 (did not apply to me at all) to 3 (applied to me very much or most of the time). The DASS-21 yields separate depression, anxiety and stress subscale scores (scores range from 0–21), and a composite total score that is the sum of the three subscales (range 0–63). Although cut-off scores defining mild/moderate/severe and extremely severe have been developed, they are recommended only for clinical practice and thus for the purposes of this study, continuous scores were used.

### Physical Activity

Physical activity was assessed using the short form of the International Physical Activity Questionnaire (IPAQ-short), which has been shown to have acceptable test-retest reliability (Spearman’s rho = 0.76) and criterion validity (Spearman’s rho = 0.30), comparable to other self-report measures [30]. Participants were asked to report the number of days in the past week and the total time per day (hours/minutes) of walking, moderate-intensity and vigorous-intensity physical activity. Data were quality controlled and total weekly physical activity was then estimated by weighting the reported minutes per week within each category (walking, moderate or vigorous activity) by a MET energy expenditure estimate assigned to each level of activity (3.3 –walking; 4.0—moderate; 8.0—vigorous) to obtain MET-minutes per week (MET.min.wk^-1^). The IPAQ-S also provided data about sitting exposure.

### Statistical Analysis

Latent class analysis was used to estimate trajectories of TV watching using an ordinal logit model, which was most appropriate for the ordinal nature of the indicator variables, TV watching at 5, 8, 10, 14, 17 and 20 years. A series of models with 1 to 6 classes were estimated, with sex used as an active covariate. To avoid local rather than global latent class solutions, each model used 200 random starts and 100 iterations per set of start values. As there are no definitive decision criteria for the optimal number of classes, the judgement as to the optimum solution was based upon a combination of statistical criteria, parsimony and interpretability [31]. The following were considered: i) the minimum values of the goodness of fit measures Bayes Information criteria (BIC), Akaike’s information criteria (AIC) and the Consistent AIC (CAIC) as indicators of the optimal number of classes, ii) consideration of the identification of the model in terms of the proportion of random starts converging on the same solution, iii) bootstrapped p-value for the log-likelihood difference between models where differences in BIC and AIC were similar, iv) the degree to which the trajectory classes identified captured distinct and potentially meaningful patterns in the data, and v) the quality of the model in terms of posterior probability diagnostics, namely the entropy R^2^ value, average posterior probability for each trajectory class, odds of correct classification and classification error. Participants were assigned to the trajectory class for which they had the highest posterior probability of membership.

General linear models were used to investigate the association of membership of TV watching class with body fat percentage with separate models for males and females, with all analyses weighted according to probability of membership for assigned TV watching class. Models were estimated unadjusted and adjusted for physical activity scores (MET.min.wk^-1^), which were log transformed due to the non-normal distribution of the data. Negative binomial regression was applied to investigate associations between TV watching classes and DASS-21 scores due to the highly skewed DASS-21 scores. A negative binomial regression reports an incident rate ratio (IRR), interpreted as the predicted proportional difference in DASS-21 scores associated with being in a particular class of TV watching. Models were estimated unadjusted and adjusted for body fat percentage and physical activity. A p value of less than 0.05 was considered statistically significant. Statistical analyses were performed using LatentGold Version 4.5 (Statistical Innovations Inc, Belmont MA USA) and SPSS v22 (SPSS Inc. Chicago, IL).

## Results

The trajectories of TV watching were estimated using all available data, i.e. 2411 participants with one or more time point (over fifteen years) reporting hours spent watching TV per week. Numbers of female and male participants for whom data were available at each time point are shown in S2 Table.

**Fig 1:**
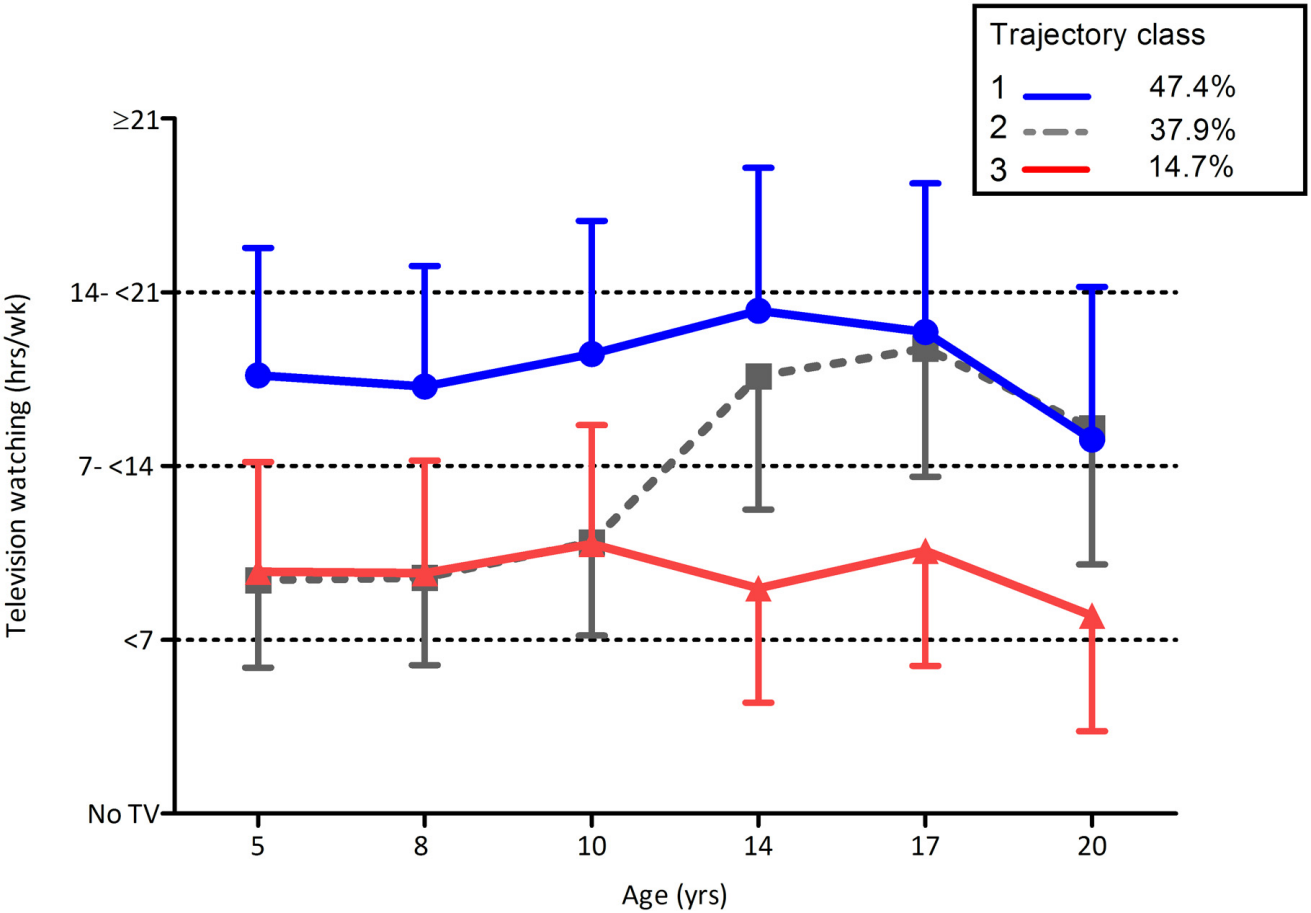
Mean television watching hours/wk by trajectory classes (Latent classes based on 15 years of TV watching data). Class 1 (blue circle); Class 2 (dashed line, grey square) and Class 3 (red triangle).

Participants were classified into three classes based on their pattern of TV watching over a 15 year time span (Fig 1). Class 1 (n = 1142 of 2411, 47.4%) was characterised by consistently high (>14 hrs/wk) levels of TV watching over the 15 years, Class 2 (n = 913 of 2411, 37.9%) was characterised by a sharp increase in TV watching over the adolescent years and Class 3 (n = 356 of 2411, 14.7%) had consistently lower (<14 hrs/wk) levels of TV watching over the course of 15 years. Sex was significantly associated with class membership (p < .001), with females estimated to comprise 45%, 47% and 59% of Class 1, 2 and 3 respectively.

For further analysis, only those cases with body composition, DASS and physical activity scores at age 20 were used (n = 910). The average probability of class membership for these cases was 0.85 (SD = 0.17) for Class 1, 0.80 (SD = 0.16) for Class 2 and 0.78 (SD = 0.20) for Class 3. Descriptive statistics of the male and female participants in the three classes at age 20 are reported in Table 1.

**Table 1:** Descriptive characteristics of participants within latent classes. Data are mean (SD) or *median (IQR) (n = 910).

	Class 1			Class 2			Class 3		
	Female	Male	All	Female	Male	All	Female	Male	All
**N**	170	213	383	153	175	328	137	62	199
**Weight (kg)**	68.2 (18.2)	79.4(15.5)	74.4 (17.7)	67.4 (17.0)	75.2 (14.5)	71.6 (16.2)	65.3 (12.2)	79.6 (14.5)	69.8 (14.5)
**Height (cm)**	165 (7)	178 (7)	172 (10)	166 (6)	178 (9)	173 (9)	166 (6)	180 (7)	171 (9)
**Lean body mass (g)**	36330 (5766)	57685 (8469)	48207 (12937)	37163 (4869)	56185 (7893)	47312 (11597)	37514 (4780)	58138 (8113)	44188 (11960)
Physical Activity (MET-mins/wk)*	2229 (957–4158)	4400 (1908–8372)	3210 (1221–6648)	2453 (1050–4002)	3546 (1872–6600)	2785 (1440–2785)	2466 (1236–4168)	3434 (1339–7200)	2564 (1280–4878)
Sitting time(hrs/wkday)*	5 (4–8)	5 (3–7)	5 (3–8)	6 (4–8)	5 (3–7)	5 (4–7)	5 (3–7)	5 (3–8)	5 (3–8)
**Body fat (%)**	41.5 (9.6)	22.7 (9.4)	31.1 (13.3)	39.2 (9.5)	20.6 (8.8)	29.3 (13.0)	38.1 (8.4)	21.0 (8.6)	32.8 (11.6)
DASS-21 Total Score *	9 (5–16)	6 (3–13)	8 (4–15)	10 (4–17)	6 (3–12)	8 (3–16)	10 (4–15)	6 (4–12)	9 (4–15)
**DASS-21 Depression sub scale**	3 (1–6)	2 (0–5)	2 (1–5)	2 (1–6)	2 (0–4)	2 (1–5)	2 (1–6)	2 (1–4)	2 (1–5)
**DASS-21 Anxiety sub scale**	2 (1–4)	1 (0–3)	2 (0–4)	2 (1–4)	2 (1–3)	2 (1–4)	2 (0–4)	1 (0–3)	2 (0–3)
**DASS-21 Stress sub scale**	4 (2–8)	3 (1–6)	3 (1–7)	4 (2–7)	3 (1–6)	4 (1–6)	5 (2–8)	3 (1–7)	4 (2–7)

### Associations between latent class and body fat at age 20

In females, membership in Class 2 or Class 3 was associated with a significantly lower percentage of body fat at age 20, compared to Class 1 before (p<0.001) and after (p<0.001) adjustment for physical activity (Table 2). For males, membership in Class 2 was associated with a significantly lower percentage body fat compared with males in Class 1 before (p = 0.045), and after (p = 0.026) adjustment for physical activity. There was no statistically significant relationship for body fat percentage for males in Class 3 compared to Classes 1 or 2.

**Table 2:** Regression coefficients (ß) and 95% CI for percentage body fat by sex and TV class unadjusted (Model 1) and adjusted (Model 2) for physical activity. Regression coefficients represent estimated mean differences in percentage body fat between groups. Data are weighted by probability of membership.

	Percentage Body Fat			
	Females	P value	Males	P value
**Model 1**	ß (95% CI)	Overall p <0.001	ß (95% CI)	Overall p = 0.102
Class 2 vs 1	-2.820 (-4.947 to -0.722)	0.006	-1.895 (-3.713 to -0.006)	0.045
Class 3 vs 1	-4.133 (-6.277 to -2.140)	<0.001	-1.768 (-4.529 to 0.829)	0.192
Class 3 vs 2	-1.312 (-3.527 to 0.697)	0.235	0.127 (-2.727 to 2.699)	0.927
**Model 2**	ß (95% CI)	Overall p <0.001	ß (95% CI)	Overall p = 0.001
Class 2 vs 1	-2.784 (-4.944 to -0.653)	0.007	-2.082 (-3.910 to -0.222)	0.026
Class 3 vs 1	-4.065 (-6.149 to -2.052)	<0.001	-2.059 (-4.735 to 0.420)	0.125
Class 3 vs 2	-1.281(-0.928 to 3.390)	0.178	0.022 (-2.601 to 2.691)	0.987

### Associations between latent class and mental health at age 20

There was no significant association between membership of TV watching class and mental health for the total DASS score before or after adjustment for percentage body fat and physical activity in either males or females (Table 3), nor for any of the subscales (model data not shown, descriptive data in Table 1).

**Table 3:** Negative binomial model risk ratios and 95% confidence intervals (95% CI) for the effect of TV classes on DASS-21 total scores by sex unadjusted (Model 1) and adjusted (Model 2) for percentage body fat and physical activity. Data are weighted by probability of membership.

	DASS-21			
	Females	P value	Males	P value
**Model 1**	RR (95% CI)	Overall p = 0.787	RR (95% CI)	Overall p = 0.523
Class 2 vs 1	1.053 (0.852 to 1.302)	0.629	0.883 (0.706 to 1.104)	0.276
Class 3 vs 1	1.076 (0.864 to 1.342)	0.510	0.999 (0.724 to 1.377)	0.995
Class 2 vs 3	1.022 (0.814 to 1.283)	0.851	1.131 (0.811 to 1.577)	0.467
**Model 2**	RR (95% CI)	Overall p = 0.735	RR (95% CI)	Overall p = 0.515
Class 2 vs 1	1.066 (0.859 to 1.321)	0.561	0.882 (0.705 to 1.103)	0.273
Class 3 vs 1	1.088 (0.869 to 1.362)	0.461	1.000 (0.724 to 1.381)	0.998
Class 2 vs 3	1.021 (0.813 to 1.282)	0.858	1.133 (0.813 to 1.581)	0.459

## Discussion

This study used latent class analysis to identify three distinct patterns of TV watching in a cohort tracked from childhood (5 years) to early adulthood (20 years) and related these trajectories to health outcomes. The latent class method was useful for providing a heuristic summary of a categorical variable and provided a data reduction process to help identify longitudinal TV watching patterns across critical life stages. Class 1 had the highest levels of TV watching across all time points measured, represented the largest proportion of the sample, and included an equal proportion of males and females. In at least one third of the sample (Class 2), TV watching behavior did not track over time and membership in this class was characterised by a sharp increase in TV watching over the adolescent period. Class 3 was characterised by the lowest levels of TV watching across all time points, included a higher proportion of females than males and represented 15 percent of the sample.

The current analysis showed that although TV watching from childhood to young adulthood appears to be a relatively stable behavior for around two thirds of participants, not everyone tracks consistently. Similar to what has been previously reported this study showed that low and high levels of TV watching may track consistently during the early childhood period (ages 2 to 6 years) [3] and into adulthood [15]. However, LCA enabled the identification of another trajectory of TV watching (Class 2) followed by over one third of participants, characterised by a steep increase in TV watching over adolescence, a time typically associated with physical activity decline, increased sedentary behaviour, body weight changes and a higher risk for depression [32]. In the current study, these higher levels of TV watching continued into young adulthood.

Prior studies examining longitudinal patterns of TV watching have typically used analytical approaches which do not allow for the modelling of population heterogeneity, and would thus have misclassified participants in Class 2 into either consistently higher or consistently lower TV watching groups. Identification of this different class suggests that the development trajectory of TV watching is not always linear and that intervention to curb the increase in TV watching over the adolescent years could be important to avoid a consistently high pattern of TV watching in later life. In the only other study which has used LCA to describe TV watching behaviour, Kwon and colleagues also showed that for a large proportion of young people, TV watching is not a stable behaviour [25]. Consistent with Class 2 in the current study, Kwon et al. identified a trajectory with increasing TV watching over time. However, the gradient of increase in TV watching in Kwon’s study was more gradual than the rapid increase in TV watching observed over the adolescent period in the current study. Kwon et al. also described a fourth trajectory, characterised by a decrease in TV watching over time, which we did not find any evidence for. Nonetheless, the ‘instability’ of TV watching behaviour demonstrated in both this study and by Kwon et al. presents opportunities for targeted intervention. It will be important for future studies to investigate reasons for this change in behaviour, which possibly include changes in family life, parenting strategies, growing influence of peers and changes in physical activity levels. Although the trajectories described by Kwon and colleagues provide compelling evidence that TV watching is not a stable behaviour for everyone, and that there were some associations with physical activity behaviours, there was no reporting of whether TV watching trajectory membership was related to health outcomes.

The statistical approach of the current study allowed us to examine TV watching trajectories across childhood and young adulthood and illustrated that, prolonged periods of high levels of TV watching over the 15 year study period (Class 1) were associated with a higher body fat percentage in young female adults compared with those in the trajectory with consistently low levels of TV watching (Class 3). Similar to the findings of Sugiyama et al (2008), the association between body fat and TV watching in the current study was stronger and more consistent for females than for males [1]. Repeated exposure and accumulation of high levels of TV watching over time may contribute to obesity and this may start in early childhood [33]. Whilst there may be a bi-directional relationship between body fat and TV watching [23], the findings of this study lend support to prior findings from the Raine study cohort, where time spent watching TV was reported to be predictive of BMI at ages 8 and 10 years [22] and other longitudinal studies which have shown that high levels of TV watching in childhood are associated with being overweight in adolescence [21] and in adulthood [14, 16].

A key contribution of this study is the finding that membership to Class 2 (the ‘unstable’ trajectory) was associated with significantly lower body fat percentage in young adults, in spite of the increase in TV watching over the adolescent years. Proctor and colleagues examined tertiles of TV watching over childhood and found that higher levels of TV watching in early childhood (age 4) predicted higher later childhood BMI (age 11)[34]. Proctor reported that participants in the middle tertile of TV watching had intermediate gains in BMI (less than the highest tertile, but more than the lowest tertile). In the current study, the lower body fat percentage associated with being in the unstable trajectory (Class 2) was not as great as for those in the consistently low (Class 3) TV watching trajectory, but was more favourable than for those in the consistently high TV watching trajectory (Class 1).

The proposed mechanisms responsible for the relationship between TV watching and increased body fat include the influence of TV content on food choices, and the increased caloric intake through between-meal snacking [35], contrasting environmental and social contexts, [1] excessive sitting, the or the displacement of physical activity [36]. Being more physically active did not appear to attenuate the relationships described in the current study, as the body fat and TV watching relationships were not diminished after adjustment for physical activity levels at age 20 in both females and males. Another study examining participants from the Raine cohort, showed that young adults who consistently participated in organised sport over their childhood and adolescent years have more favourable health outcomes at age 20 [37]. Future analyses with these trajectory classes should assess correlates of class membership and associations with other health outcomes.

In the current study, TV watching levels over the 15 year period were not associated with symptoms of depression, anxiety or stress in young adulthood for males or females. In an earlier cross sectional analysis of the Raine study cohort (at age 14 years), greater time spent in front of a screen was associated with poorer mental health [5]. Currently, only three longitudinal studies have examined the relationship between sedentary behaviour and mental health, but data from the childhood to early adulthood period are lacking. A two-year follow up study in older adults showed that TV watching for more than six hours per day was associated with a higher risk for depressive symptoms [18]. In middle aged and older adults, an association between TV watching and a higher risk of depressive symptoms has been reported [17, 19]. However, when participants in Lucas’ (2011) study with clinically diagnosed depression were analysed separately, the association of depression with TV watching was no longer apparent [17]. Importantly, it has been shown that if a sufficient level of physical activity is reached, sedentary habits might be not as definitive in predicting mental disorders [19]. Mekary and colleagues (using isotemporal substitution in older adults) found that watching TV for 60 minutes/day instead of easy paced walking (for 60 minutes/day) was not associated with depression risk, but that watching TV for 60 minutes/day instead of brisk paced walking (for 60 minutes/day) was associated with a higher depression risk [38]. Rebar and colleagues (using a cluster analysis approach in middle aged adults) found that a cluster representative of higher psychological distress was not profiled as physically inactive [39]. It may be that the intensity of physical activity is important to offset the risk of mental health problems. Although an association between TV watching and depressive symptoms has been reported in older adults, the longitudinal relationship from childhood to adulthood remains unclear.

Only TV watching was considered in this analysis, yet many participants may have had substantial exposure to other screen based media (e-mail, instant messaging, surfing the Internet, playing electronic games) which may have influenced class membership and associations with health outcomes. The change from parent-report to child-report may have influenced the trajectories; however this is unlikely as findings were consistent using either parent- (data not shown) or child-report data at 14 years. The study did not investigate why TV watching increased for over one third of adolescents, and the findings may not be applicable to a more heterogeneous population from different geo-socio-economic backgrounds. Despite these limitations, this study had important strengths which included comprehensive analysis of data from six time point’s spanning childhood to early adulthood in a large community sample. Also, this study applied a sophisticated analytical approach enabling data driven identification of three distinct trajectories. The trajectories of TV watching were related to important health outcomes and provided clear evidence for the association of TV watching in childhood and obesity outcomes in early adulthood.

The three distinct trajectories of TV watching identified in this study indicate that there are clear opportunities to intervene during at least two critical periods of development (childhood and adolescence). Most importantly, this study identified a subset of participants with low levels of TV watching in childhood and also that this subset, despite an increase in TV watching over the adolescent years, maintained a lower level of body fat in young adulthood. Health professionals should continue to promote television watching of less than 14 hours per week, particularly in childhood and during adolescence and be cognisant of the importance of not assuming that TV watching always follows a stable trajectory.

## Supporting Information

S1 Table Comparison between those included and not included in this study. (DOCX)

S2 Table Numbers of females (F) and males (M) within TV classes at ages 5, 8, 10, 14, 17 and 20 years. (DOCX)
