# Supplementary material for: Trajectories of Television Watching from Childhood to Early Adulthood and Their Association with Body Composition and Mental Health Outcomes in Young Adults
Source: PLoS One. 2016 Apr 20;11(4):e0152879. doi: 10.1371/journal.pone.0152879 (PMC4838324; doi:10.1371/journal.pone.0152879)
Supplement: S1 Table — (DOCX) [file pone.0152879.s001.docx]

**Supplementary Tables**

S1 Table. Comparison between those included and not included in this study.

|  | **Participants in this study (n=910)** | **Yr 20 participants not included in this study (n=655)** | **P value** |
| --- | --- | --- | --- |
| Sex (% female) | 51 | 49 | #0.356 |
| BMI at age 5 (kg/m^2^) | 15.79 (1.81) | 15.84 (1.78) | 0.511 |
| BMI at age 20 (kg/m^2^) | 24.29 (5.14) | 25.01 (5.38) | 0.026 |
| High family income at 5 yrs of age (>$40000/annum) (%) | 48 | 36 | #<0.086 |
| High income at 20 yrs of age (>$500/wk) (%) | 31 | 26 | #0.068 |

# P value for Chi-square shown
