# Supplementary material for: Trajectories of Television Watching from Childhood to Early Adulthood and Their Association with Body Composition and Mental Health Outcomes in Young Adults
Source: PLoS One. 2016 Apr 20;11(4):e0152879. doi: 10.1371/journal.pone.0152879 (PMC4838324; doi:10.1371/journal.pone.0152879)
Supplement: S2 Table — (DOCX) [file pone.0152879.s002.docx]

S2 Table. Numbers of females (F) and males (M) within TV classes at ages 5, 8, 10, 14, 17 and 20 years.

|  | **5yrs** | | **8yrs** | | **10yrs** | | **14yrs** | | **17yrs** | | **20yrs** | |
| --- | --- | --- | --- | --- | --- | --- | --- | --- | --- | --- | --- | --- |
|  | n=2192 | | n=2107 | | n=2017 | | n=1599 | | n=1270 | | n=1240 | |
|  | F | M | F | M | F | M | F | M | F | M | F | M |
| **Class 1** (Consistently high TV watching) | | | | | | | | | | | | |
| No TV | 0 | 0 | 0 | 1 | 0 | 0 | 0 | 2 | 0 | 0 | 6 | 10 |
| Less than 7 hrs/wk | 25 | 28 | 26 | 22 | 21 | 35 | 15 | 14 | 17 | 17 | 43 | 58 |
| 7 to <14 hrs/wk | 206 | 262 | 220 | 287 | 127 | 196 | 75 | 94 | 72 | 97 | 114 | 124 |
| 14 to <21 hrs/wk | 190 | 242 | 161 | 193 | 203 | 222 | 157 | 209 | 117 | 102 | 75 | 71 |
| 21 hrs/wk and more | 37 | 48 | 33 | 42 | 46 | 69 | 74 | 83 | 58 | 63 | 16 | 19 |
| **Class 2** (TV watching increases over adolescence) | | | | | | | | | | | | |
| No TV | 0 | 0 | 0 | 0 | 0 | 0 | 0 | 0 | 0 | 0 | 0 | 0 |
| Less than 7 hrs/wk | 247 | 287 | 244 | 261 | 173 | 193 | 16 | 16 | 5 | 12 | 31 | 51 |
| 7 to <14 hrs/wk | 141 | 141 | 125 | 160 | 173 | 199 | 128 | 159 | 111 | 101 | 125 | 112 |
| 14 to <21 hrs/wk | 4 | 3 | 5 | 0 | 17 | 16 | 102 | 104 | 105 | 87 | 62 | 48 |
| 21 hrs/wk and more | 0 | 0 | 0 | 0 | 0 | 2 | 30 | 32 | 31 | 34 | 12 | 5 |
| **Class 3** (Consistently low TV watching) | | | | | | | | | | | | |
| No TV | 10 | 9 | 9 | 7 | 4 | 0 | 15 | 7 | 8 | 6 | 20 | 13 |
| Less than 7 hrs/wk | 125 | 60 | 130 | 62 | 110 | 58 | 110 | 77 | 65 | 39 | 117 | 42 |
| 7 to <14 hrs/wk | 69 | 45 | 55 | 44 | 82 | 47 | 52 | 19 | 80 | 32 | 37 | 24 |
| 14 to <21 hrs/wk | 9 | 3 | 14 | 6 | 12 | 10 | 6 | 2 | 5 | 6 | 4 | 1 |
| 21 hrs/wk and more | 0 | 1 | 0 | 0 | 2 | 0 | 1 | 0 | 0 | 0 | 0 | 0 |
